# Trends of Selective Fetal Reduction and Selective Termination in Multiple Pregnancy, in England and Wales: a Cross-Sectional Study

**DOI:** 10.1007/s43032-021-00819-5

**Published:** 2021-12-13

**Authors:** Sreya Sam, Sarah Tai-MacArthur, Panicos Shangaris, Srividhya Sankaran

**Affiliations:** 1 GKT School of Biomedical Sciences, Kings College London, Guy’s Campus, Great Maze Pond, London, SE1 1UL UK; 2 School of Bioscience Education, Kings College London, Guy’s Campus, Great Maze Pond, London, SE1 1UL UK; 3 Department of Women and Children’s Health, School of Life Course & Population Sciences, Faculty of Life Sciences and Medicine, Kings College London, London, 10th Floor North Wing St Thomas’ Hospital, London, SE1 7EH UK; 4 Department of Women and Children, Guy’s and St Thomas’ NHS Foundation Trust, London, SE1 7EH UK

**Keywords:** Selective abortion, Selective fetal reduction, Selective termination, Multiple pregnancy

## Abstract

Selective abortion was shown to be increasingly common in England and Wales over a 9-year period, occurring most frequently as twin to singleton reductions in the 1st trimester. We analysed the trends in selective abortion (SA) in multiple pregnancies in England and Wales between 2009 and 2018. This is a cross-sectional study looking at 1143 women with multiple pregnancies in England and Wales undergoing SA. There were a total of 1143 cases of SA between 2009 and 2018 in England and Wales, representing 0.07% of total abortions. There has been a steady increase in cases, from 90 in 2009 to 131 in 2018, with 82.3% justified under ground E of The Abortion Act 1967. The majority of SAs were carried out at 13–19 weeks gestation, and intracardiac injection of potassium chloride was the most prevalent method (75%). Twin to singleton reductions accounted for 59%, the most common form of SAs. Over half of all cases (59%) were performed in women aged 30–39 years, and 84% of all women were of White ethnicity. SA has been an option available for couples diagnosed with multiple pregnancy, especially when there are discordant anomalies. Although SA may decrease multiple pregnancy-related complications, preventative methods must be championed.

**Supplementary Information:**

The online version contains supplementary material available at 10.1007/s43032-021-00819-5.

## Introduction

Multiple pregnancy, defined as a pregnancy in which more than one fetus develops, accounts for approximately 3% of all live births worldwide [1, 2]. The incidence of multiple pregnancies in England and Wales has risen in the last 30 years, from 10 in 1000 in 1980 to 16 in 1000 in 2016 [3]. In 2018, there were a total of 10,005 multiple births in England and Wales, of which 99% were twins and 1% triplets [4].

This uptick in cases may be attributed to the rise in assisted reproductive technologies (ART), which has been shown to predispose to multiple pregnancies [5]. The Human Fertilisation and Embryology Authority (HFEA) register data showed that 23% of women who have undergone in vitro fertilisation (IVF) would have twins, compared to only 2% in the general population [6]. The incidence of monozygotic twins is estimated to increase threefold after ovulation induction or conventional IVF and 13-fold after intracytoplasmic sperm injection [7]. This increasing incidence of multiple pregnancies may be of concern due to their association with high-risk complications [8].

Multiple pregnancies are associated with pre-term birth, structural abnormalities, maternal morbidity and fetal mortality [8]. Pre-term delivery is one of the most recognised complications, and its related sequelae correlate with the fetal number [9]. Moreover, babies born from multiple births tend to have lower birth weights than singletons and carry a higher risk of stillbirth, infant death and child disability [3]. Monochorionic twins have specific complications such as twin-to-twin transfusion syndrome, which, if not treated, can be fatal. In addition, women with multiple pregnancies are more likely to experience maternal complications such as pregnancy-induced hypertension, pre-eclampsia, gestational diabetes, anaemia and haemorrhage [10].

Selective fetal reduction (SFR) refers to reducing a higher-order pregnancy by removing one or several fetuses, usually performed in the first trimester to maximise positive outcomes for the remaining fetus. Current evidence suggests that SFR can prolong pregnancy, thereby reducing prematurity, increasing birthweight and maximising positive outcomes for the remaining fetus(es) [11]. This is particularly relevant in the face of a diagnosis of a severe congenital abnormality—the affected fetus could be terminated to improve the viability of the unaffected fetus. This scenario is often referred to specifically as selective termination (ST).

The legal status of SA was clarified through the HFEA Act of 1990, which allows for SA in circumstances that satisfy The Abortion Act 1967. Thus, if the mother has multiple high-risk pregnancies or a fetal diagnosis of a severe anomaly, SFR/ST may be lawful under Sect. 1(1)a, (b) or (c) of the Act [12]. Though protected legally, there is still discourse occuring around the ethical considerations of SA. SA is typically performed using intracardiac potassium chloride (KCl) injection, though vasoocculsive techniques like radiofrequency ablation (RFA), bipolar cord coagulation (BCC) or interstitial laser ablation (ILA), which can be used in monochorionic twins when intracardiac KCl is unsuitable.

### Aims and Objectives

This study aims to identify and analyse trends of SFR/ST in multiple pregnancies in residents and non-residents of England and Wales between 2009 and 2018.

## Materials and Methods

We conducted a retrospective observational study examining the trends of SFR/ST after multiple pregnancies in England and Wales over the last 9 years. Data was provided by the Department of Health and Social Care (DHSC). They combine both SFR/ST and describe this globally as ‘selective abortion’ (SA). We analysed trends in maternal age, ethnicity, gestation, legal grounds and original number of fetuses.

### Study Population

We collected data from the Health and Social Act 4 (HSA4) forms submitted by clinics and hospitals to the DHSC. The DHSC collated aggregated data on abortion statistics relating to SA in multiple pregnancies performed in residents and non-residents in England and Wales between 2009 and 2018. All women, including residents and non-residents, who underwent SA within this period were included. No raw patient-level data was used, and therefore we did not require approval from the Chief Medical Officer (CMO). A decision tool completed on the NHS Health Research Authority website confirmed that no approval was needed from the Research Ethics Committee. The data was then analysed to identify trends.

Data from 2012 is excluded in this study as data variables were incomplete and unavailable to be extracted. Similarly, data on the method of fetocide is not available for non-residents of England and Wales in 2013 and hence were not included in this analysis. Additionally, due to a small total number of fetocides and disclosure risks associated with this, we could not acquire data on the details of the chorionicity of the higher-order pregnancies. Due to confidentiality concerns, the post-reduction number of fetuses was not reported in pregnancies of 4 fetuses or above.

## Results

### Prevalence

There were a total of 1,753,074 abortions carried out between 2009 and 2018 (excluding 2012), in England and Wales, with 1143 of them being cases of SA. SAs were found to be relatively rare, representing 0.07% of the total number of abortions within our study period.

**Fig. 1:**
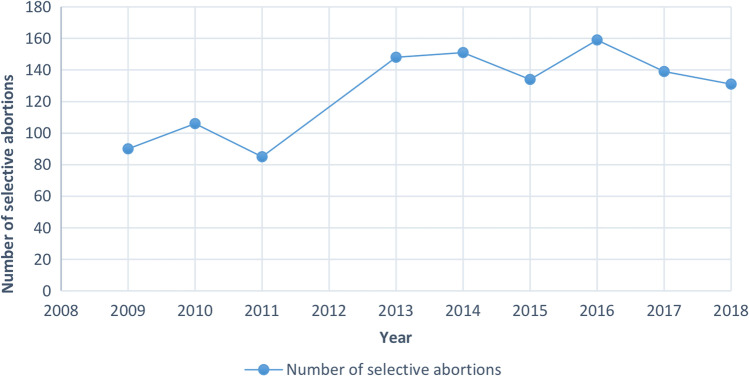
Prevalence of selective abortions in England and Wales (2009–2018). Total number of SAs performed in residents and non-residents in England and Wales each year between 2009 and 2018

Figure 1 shows a steady increase in cases of SAs over the last 10 years, with an average annual increase of 4.6%. The total number of cases has increased by 45.6%, from 90 cases in 2009 to 131 cases in 2018.

### Statutory Grounds

The majority of SAs were conducted under statutory ground E, which states, ‘if the child were born, there is a substantial risk that it would suffer from serious physical or mental abnormalities’. Statutory ground E is usually used in cases where there is a congenital abnormality. A total of 940 SAs (82.3%) were categorised as ground E either alone or in combination with A, B, C or D (Supplementary Fig. 1). Ground C (alone) was reported in 187 cases (16.3%), the subsequent highest number. Abortion is lawful under ground C if the pregnancy has not exceeded its 24th week, and the continuation of the pregnancy would involve a risk greater than if the pregnancy was terminated or injury to the physical or mental health of the pregnant woman. Grounds A, B or D were limited to few cases (1.3%) over this 10-year period.

### Method of Fetocide in Multiple Pregnancy

**Fig. 2:**
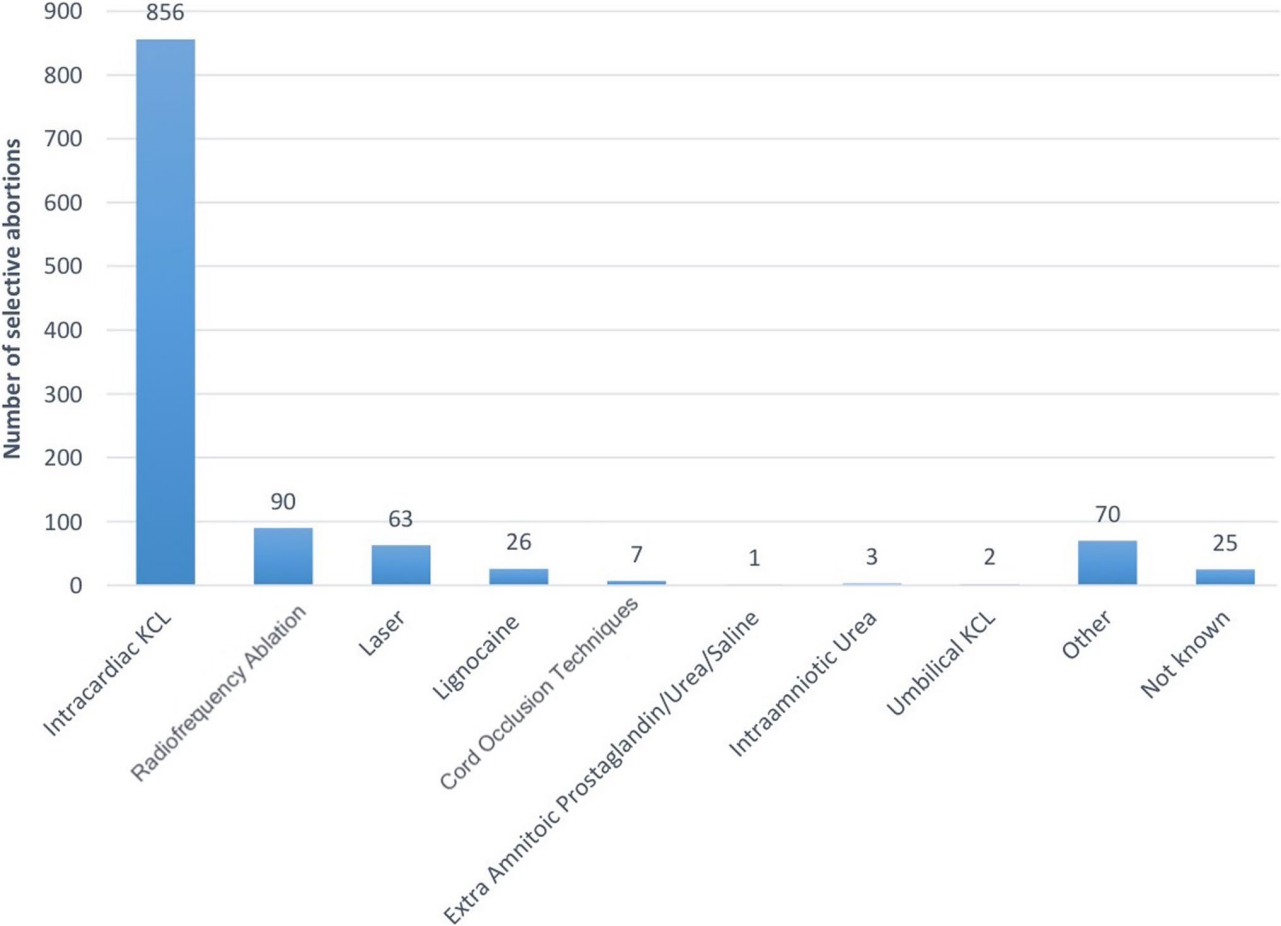
Method of fetocide in England and Wales (2009–2018). Prevalence of different methods of SA in residents and non-residents in England and Wales between 2009 and 2018

Cumulatively, the majority of all SAs were carried out by an intracardiac injection of potassium chloride (KCL), accounting for 75% of all cases of SA from 2009 to 2018, as shown in Fig. 2. No patient-level demographic data or chorionicity were accessible for these cases. RFA was the next most common, accounting for 8% of cases, followed by ILA 6%, lignocaine 2% and BCC 0.6%. Eight percent of cases were either not known or categorised as other. There were few cases of extra amniotic prostaglandin/urea/saline, intra-amniotic urea and umbilical KCL.

### Original Number of Fetuses

**Fig. 3:**
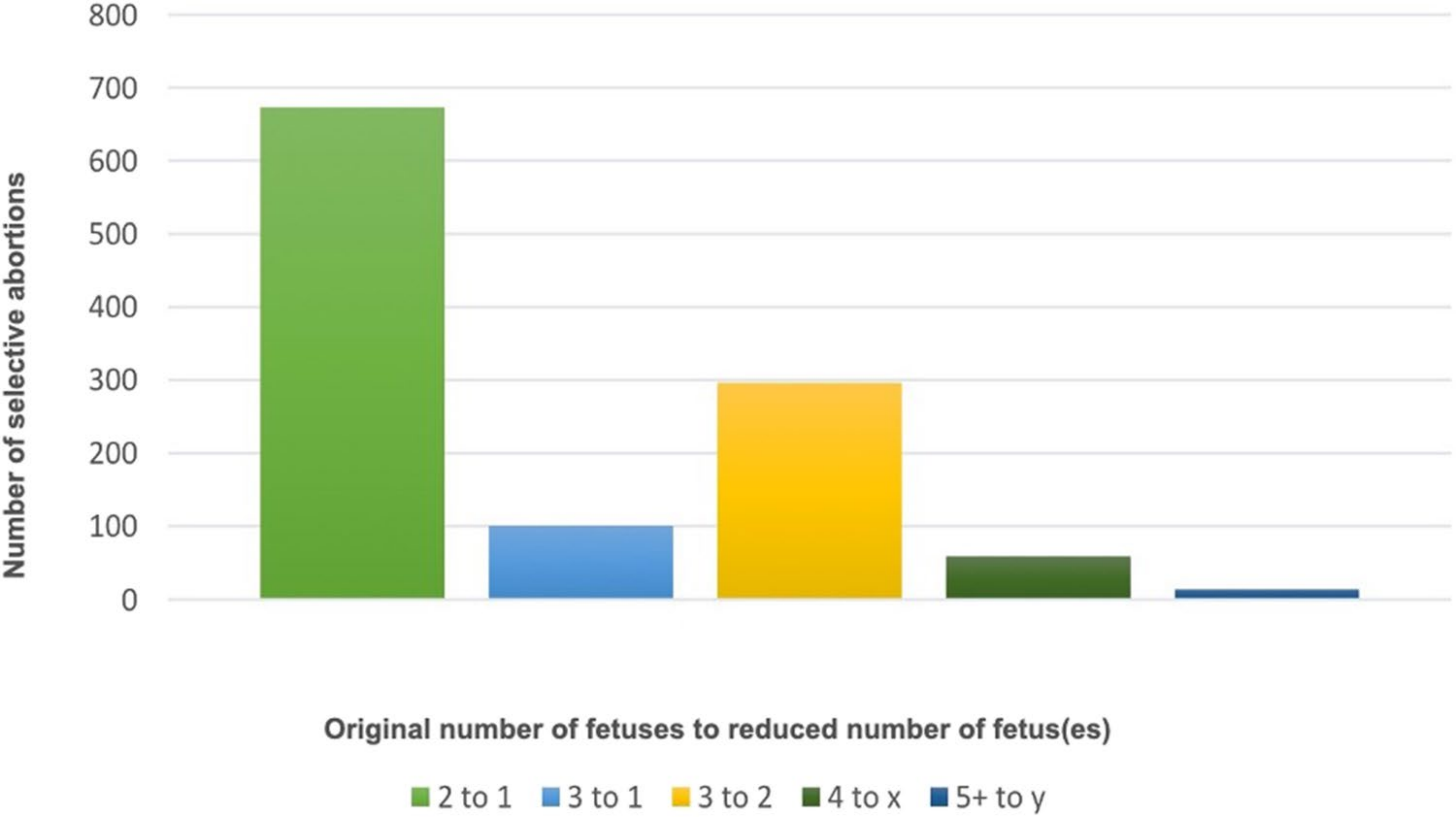
Selective abortions by original number of fetuses in England and Wales (2009–2018). Total number of SAs performed grouped by original number of fetuses to number of fetus(es) post reduction, in residents and non-residents in England and Wales between 2009 and 2018. For example, a twin to singleton reduction is described as 2 to 1

We found that 83% of SAs were conducted on twins reduced to a singleton pregnancy (2 to 1), as shown in Fig. 3. Triplet to twin (3 to 2) accounted for 296 cases (26%). There were 101 triplets to singleton (3 to 1) cases of SAs (9%). SA was carried out in higher-order pregnancies of 4 or 5 or more, although due to disclosure concerns, the number of the fetus(es) post-reduction is unknown. Post reduction numbers of these pregnancies are represented as *x* or *y*, respectively. A reduction from 4 to *x* accounted for 59 cases (5%), and 5 to *y* accounted for 14 cases (1%).

### Gestation

**Fig. 4:**
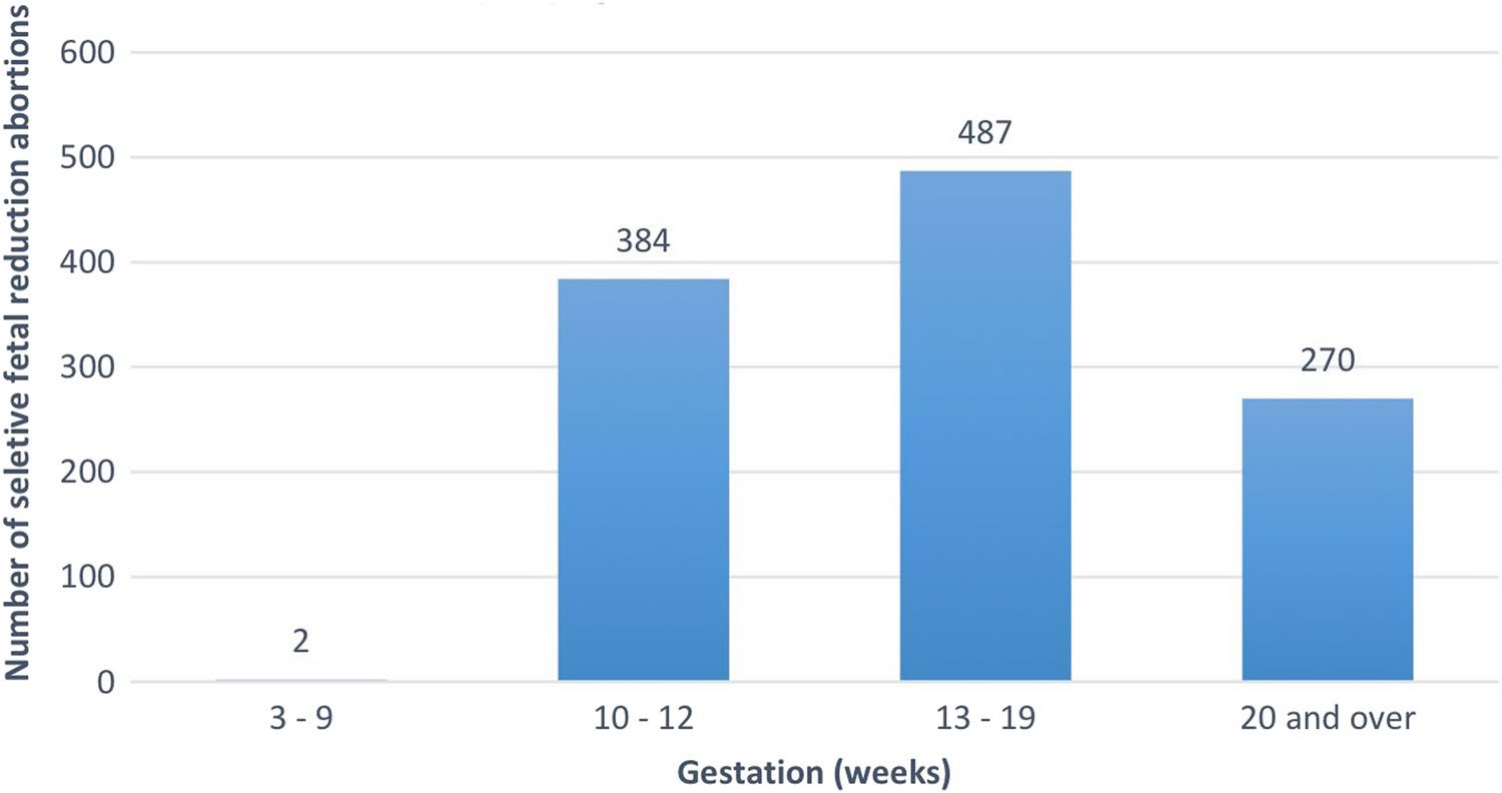
Prevalence of selective abortions by gestation in England and Wales (2009–2018). SAs grouped by gestational age in weeks in residents and non-residents of England and Wales between 2009 and 2018

The most common gestation to undergo SA was 13–19 weeks, the second trimester as shown in Fig. 4. In total, 42.6% of SA cases were carried out during this time, a total of 487 cases. A third of cases (33.8%) were performed in the first trimester, with 384 cases occurring between 10 and 12 weeks gestation and only two between 3 and 9 weeks. A further 270 cases took place after 20 weeks accounting for 23.6%.

### Maternal Age

Maternal age was presented in ranges for confidentiality. In addition, the data was aggregated and, therefore, not able to be interpreted over time. The mode maternal age range was 30–34 years with 340 cases (Supplementary Fig. 4). Following closely behind, 329 cases were carried out in women aged 35–39 years. Combined, women aged 30–39 years account for 59% of the total population. The least prevalent age range was women less than 20 years old.

### Ethnicity

The majority of women who underwent SAs were of white ethnicity, the largest proportion of which being White British, accounting for 59% of the total population studied (Supplementary Fig. 5). A further 243 (21%) cases were white (any other background), and 43 (4%) were White Irish (Supplementary Fig. 6). Nine percent were Asian or Asian British, 3% were Black or Black British, 1% Chinese, 1% were of mixed background. In addition, 2% were either labelled as other or not known/stated. Indian women accounted for 55% (59 cases) of the Asian or Asian British population. However, this is only 5% of the total women who underwent SA over the time period (Supplementary Fig. 7).

## Discussion

This data suggests that although SA remains relatively rare, its incidence in England and Wales has increased over the last 10 years. This is in spite of cases of multiples decreasing over the same study period [13]. This may be attributed to better anomaly detection on prenatal scans, hence SFR being offered more frequently as an option in the management of multiple pregnancies with discordant anomaly or growth. SA cases saw a peak in 2016, with a subsequent decline since. Although the incidence of multiple pregnancy remains high, a decrease has been seen in England and Wales for the third consecutive year, beginning in 2016, correlating well with our data [3]. This may be due to preventative methods such as elective single embryo transfer (eSET) after IVF treatment and controlled ovulation stimulation.

Assessment of the statutory grounds for SA clarifies the personal and clinical decision-making process—the large majority of SA cases were conducted under ground E or including ground E, suggesting that fetal anomaly is a key deciding factor in SA. The incidence of fetal abnormalities has been higher in multiple pregnancies than singletons [14]. Another retrospective observational study looking at 88 SFRs, found that the leading indication for SFR in both monochorionic and dichorionic twins was central nervous system abnormalities, which corroborates our data [15].

Though methods of fetal reduction have evolved alongside an expanding evidence base, this study shows that KCl injection is still the commonest method. KCl injection can only be safely used in dichorionic diamniotic (DCDA) multiples. Although chronicity is not recorded on the HSA4 form, studies suggest that DCDA multiples are the commonest in the UK, which may explain the dominance of KCl injection [16].

The most common type of SA that took place in our study period was twin to singleton reductions. This is of interest due to the debate on the clinical benefit of this procedure, partially based on the perception that twin pregnancies already have generally favourable outcomes and therefore would not require intervention. Evans et al. found that twin to singleton reduction reduced the likelihood of miscarriage by 5% [17]. Multiple studies suggest a significant reduction in the risk of pre-term birth and prematurity even when adjusting for parity and mode of conception [18, 19]. Outcomes may be confounded by the grouping of elective SFR and ST for fetal anomaly. Haas et al. [20] and Vieira et al. [21] investigated purely elective SFR. Both found elective twin to singleton reduction resulted in lower odds of preterm delivery and low birth weight. Additionally, Vieira et al. found a reduction in the odds of developing pre-eclampsia, preterm premature rupture of membranes and caesarean delivery [21]. Alternatively, Hasson et al. observed no improved pregnancy outcomes in twin to singleton reduction; however, most cases included were ST for fetal anomaly, and occurred late in pregnancy (15 weeks) [22]. Luo et al. report a higher total miscarriage rate in elective twin reductions, and therefore a reduced chance of taking home a live baby when compared to non-reduced twin pregnancies [23].

The decision to reduce twins to a singleton is therefore complex and strikes a balance between medical need and maternal autonomy. In recent years, the demographics of pregnant women are changing, with more multiple pregnancies occurring in older women, particularly in those who conceived via ART. For some women, psychological and socio-economic factors play a significant role in reducing twins to a singleton compared to medical factors. Evans et al. predict that as societal norms evolve, the ethical debate will grow around SFR in those contexts [17]. The ethical concerns surrounding SFR frequently overlap with those raised in discussions around the ethical basis of abortion—opponents with ‘pro-life’ viewpoints are unlikely to support SFR. However, even some ‘pro-choice’ ethicists see abortion and SFR as morally distinct, specifically questioning the validity of twin to singleton reduction [24]. Dahl et al. refute the arguments presented against SFR, concluding a morally significant difference between SFR and other abortions cannot be demonstrated, and therefore in a society where abortion is available and morally acceptable SFR requires no additional justification [25]. Both termination of pregnancy and SFR patient centre autonomy and are protected in the UK under the Abortion Act, making it ultimately the woman’s right to choose. A full discussion of the medical ethics discourse surrounding SFR is beyond the scope of this paper and the reader is referred to the literature for further details [25–29].

Beyond twin to singleton reductions, it is clear that the survival benefits are most dramatic in high order pregnancies of 4 or 5 [28, 30]. The low and decreasing rate of SA in high order pregnancies of 4 or 5 + potentially speak to preventative methods limiting multiple pregnancies, as the rate of SA in pregnancies with four or more fetuses has constantly stayed low from 2011 to 2018, with the lowest ever numbers seen in 2018 [3].

Our data suggests most SA occurred in the second trimester at 13–19 weeks, in contrast with non-selective abortions, which generally occur in the first trimester. Although the first trimester is considered a safe period for SFR, there is some debate amongst researchers about the optimal weeks within this time frame. Tadin et al. suggest that 12–14 weeks is the safest period and allows a better prognosis for the remaining fetus(es) [31]. Equally, evidence advocates a safe period as low as 10 weeks or as high as 15 weeks [30, 32]. Our data showed that 33.5% of SAs were carried out between 10 and 12 weeks gestation. Importantly, termination due to severe fetal anomaly has no gestational age limit. Ground E was by far the most prevalent indication for SA in our study. Therefore, it is feasible that the SA cases after 13 weeks were likely due to fetal anomaly detection. Most screening for anomaly (i.e. amniocentesis or routine anomaly scans) occurs post 13 weeks. Notably, the grounds for termination and the gestational age were not directly correlated or compared.

The peak age of women who had SA between 2009 and 2018 was between 30 and 34 years, followed by 35–39 years. This age group accounted for 59% of the total population of women in England and Wales who had a SA in that time period. Women of 30–39 years occupy the majority of the IVF population, accounting for 79% in 2016 [6]. The high prevalence of IVF use in that population may explain the high incidence of SA. The higher frequency of SA in an older age range could be attributed to a societal shift, in which, increasingly, women have children later in life, both using ART or conceiving naturally. It is documented that children of older mothers are at an increased risk of certain congenital abnormalities and therefore may choose SA [33].

The vast majority of women included in this study were Caucasian. Statistics on non-selective abortion also report a similar majority Caucasian demographic. Some explanations for lower SA rates in minority ethnic groups include lower ART use—a US study showed a disparity in the use of ART amongst Black women (4.6%), compared to White women (85.6%) between 1999 and 2000 [34]. However, evidence suggests an increasing prevalence of infertility amongst Black women, from 7.8% in 1982 to 11.6% in 2002 [35]. It is unclear whether these differences reflect access to treatment or attitudes towards ART. The figures seen in this study correlate with our data on the rates of SA. If fewer Black women are using ART, they inadvertently minimise the risk of iatrogenic multiple pregnancies and subsequent need for SA.

## Conclusion

This was a large-scale study using national data from the DHSC archives to identify trends in SA across England and Wales over 10 years. The prevalence of multiple pregnancies and their associated complications has drastically increased since the rise of ART. SFR has been developed as a solution to try and minimise these risks for both mother and baby. Additionally, ST can be carried out on a fetus diagnosed with a severe abnormality in utero, whilst the remaining fetuses are healthy. Though still relatively uncommon, the personal and clinical decision-making behind SA is complex, and this data provides greater insight into demographics, methods and indications.

The limitations of this study include its retrospective design. In addition, there were confidentiality concerns leading to unreported outcomes like the number of fetuses reduced in 4 + multiple pregnancies. HSA4 forms also do not collect data on chorionicity, which is essential for deciding the method of SA; therefore, we can only speculate about indications for various management options. As an observational study, no causal relationships between factors affecting SA could be made. Also, only outcomes such as complications are collected; hence, no information is available about the outcome of delivery such as gestational age at delivery and livebirth status. Further research is required to analyse these trends to a greater extent.

The data submitted via the HSA4 form to DHSC is rich in scientific data such as indications, methods and gestational age, of termination of pregnancy. It would be desirable to collect more outcome data, which will make it a robust national registry of cases of medical terminations of pregnancy. As multiple pregnancy only contributes to a small proportion of pregnancies that continue beyond the termination of a single fetus, it would not be too onerous to collect this information. Hence, we recommend updating the form in consultation with fetal medicine experts to make it relevant to the times. Not many countries can boast of such a comprehensive registry, and hence, it can set precedence internationally.

SA has been accepted as a safe method of reducing the risks of multiple pregnancy. Regardless of this, the prevention of higher-order pregnancies should remain the focus of health intervention. However, complete avoidance is unrealistic and spontaneous multiples will continue to occur. Therefore, SA continues to be a reasonable option. It is crucial to find the optimal conditions for SA and identify trends that could influence clinical practice in the UK.

## Supplementary Information

Below is the link to the electronic supplementary material.

Supplementary file1 (DOCX 1035 KB)

## Data Availability

Data subject to third party restrictions. The data that support the findings of this study are available from The Department of Health and Social Care. Restrictions apply to the availability of these data, which were used under licence for this study. Data are available from the authors with the permission of The Department of Health and Social Care.
