# Supplementary material for: Trends of Selective Fetal Reduction and Selective Termination in Multiple Pregnancy, in England and Wales: a Cross-Sectional Study
Source: Reprod Sci. 2021 Dec 13;29(3):1020–7. doi: 10.1007/s43032-021-00819-5 (PMC8863756; doi:10.1007/s43032-021-00819-5)
Supplement: Supplementary file 1 — Supplementary file1 (DOCX 1035 KB) [file 43032_2021_819_MOESM1_ESM.docx]

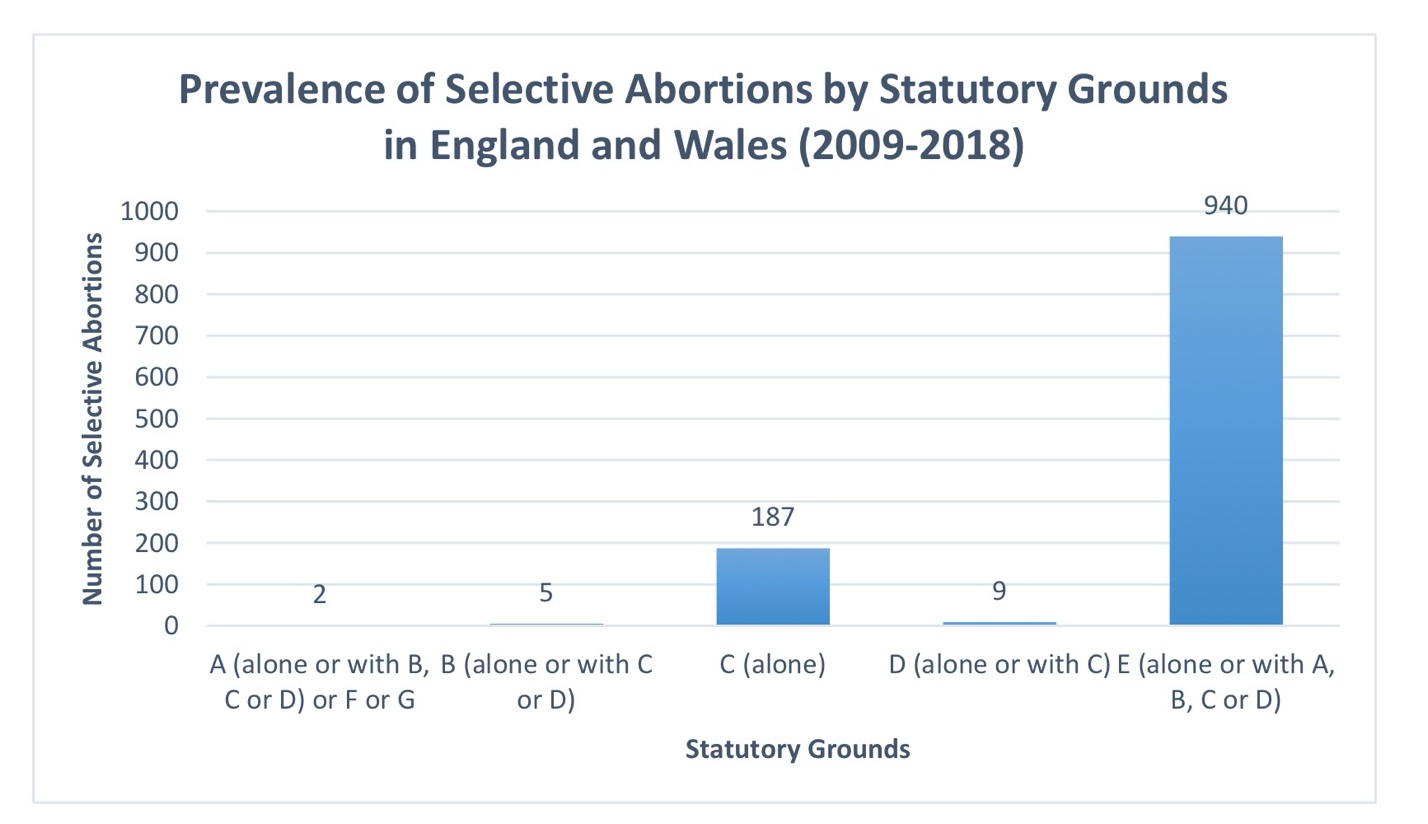


**Supplemental Figure 1.** Prevalence of Selective Abortions by Statutory Grounds in England and Wales (2009-2018)

This graph depicts the prevalence of SA by Statutory Grounds of the Abortion Act 1967 in residents and non-residents in England and Wales between 2009- 2018.

**
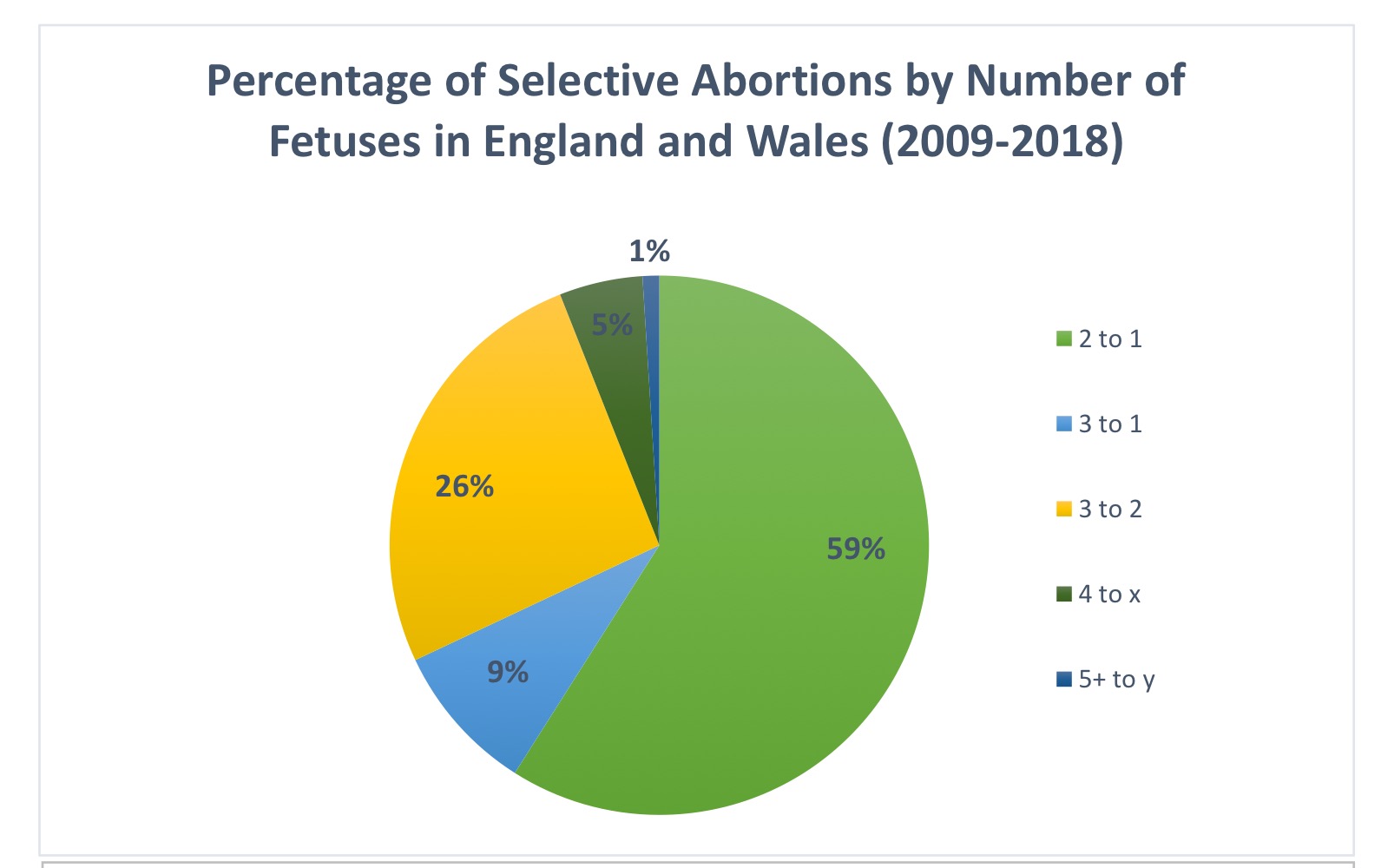
**

**Supplemental Figure 2.** Percentage of Selective Abortions by Number of

Fetuses in England and Wales (2009-2018.)

This graph depicts the percentage of SA by the number of original fetuses to the number of fetus(es) post reduction in residents and non-residents in England and Wales 2009-2018.


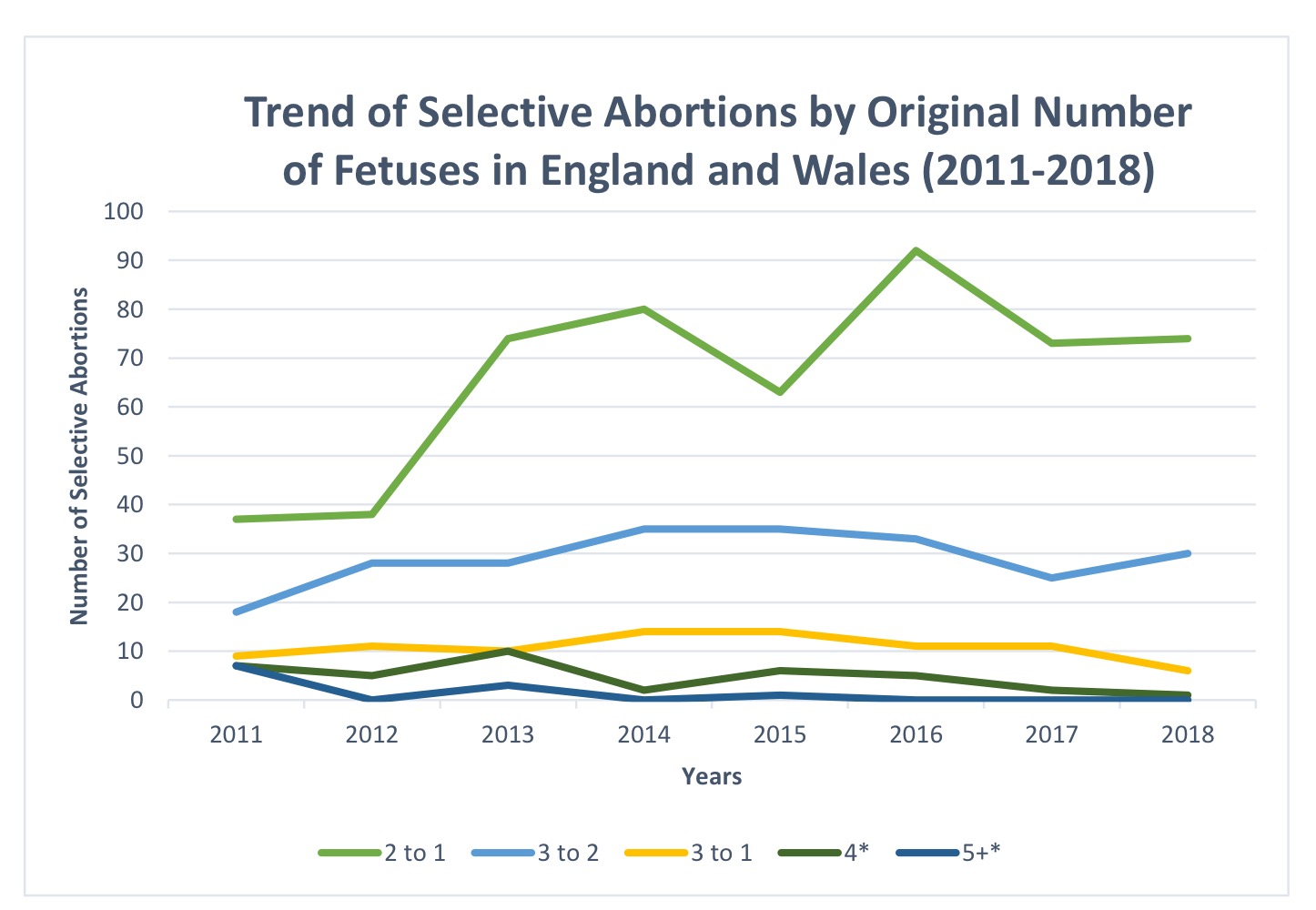


**Supplemental Figure 3.** The trend of Selective Abortions by Original Number of Fetuses in England and Wales (2011-2018)

This graph depicts the trend of SA by the number of original fetuses to the number of fetuses post-reduction in residents and non-residents in England and Wales 2011-2018. Data from the DHSC published Abortion Statistics report


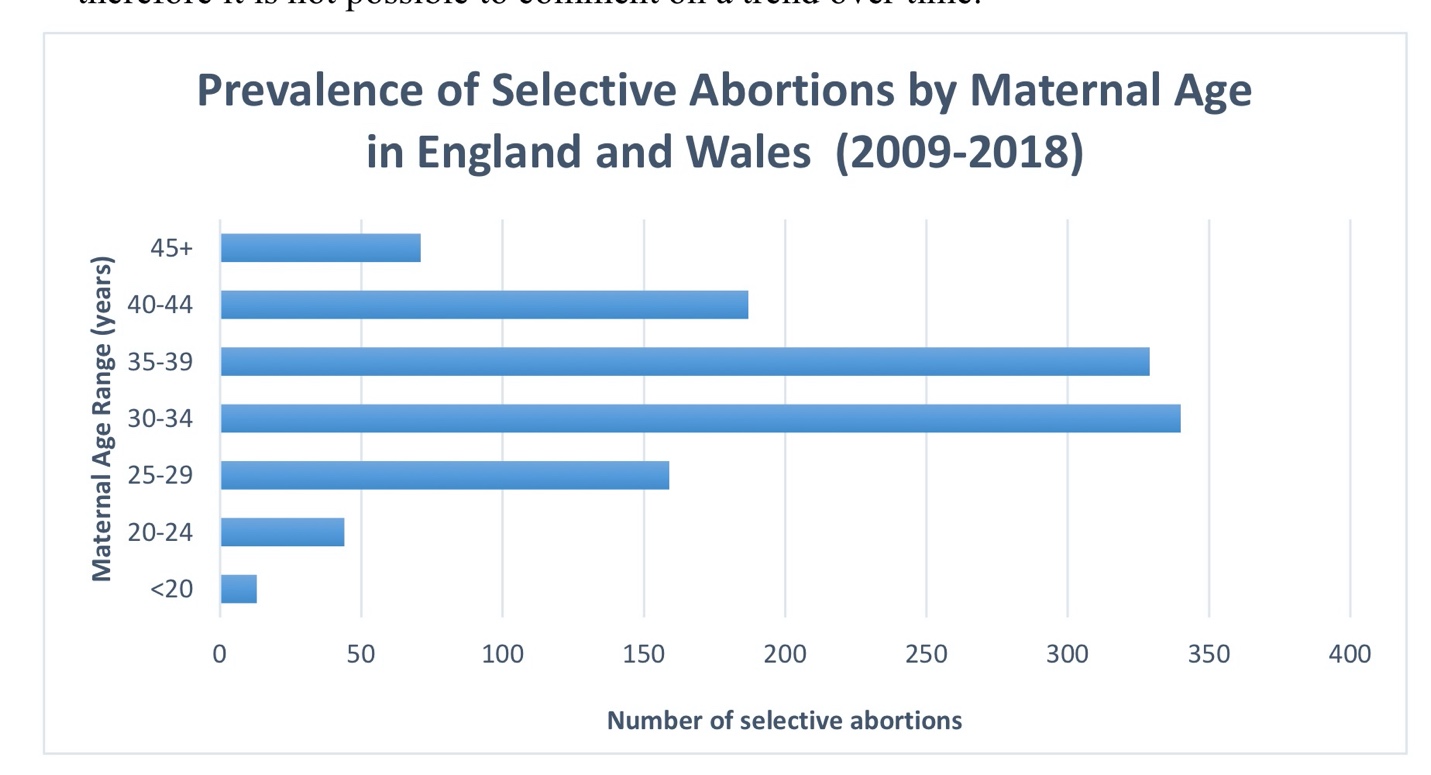


**Supplemental Figure 4.** Prevalence of Selective Abortion by Maternal Age in England and Wales 2009-2018

This graph depicts the prevalence of SA by maternal age in residents and non-residents of England and Wales between 2009-2018. Maternal age is categorised into age ranges (years).


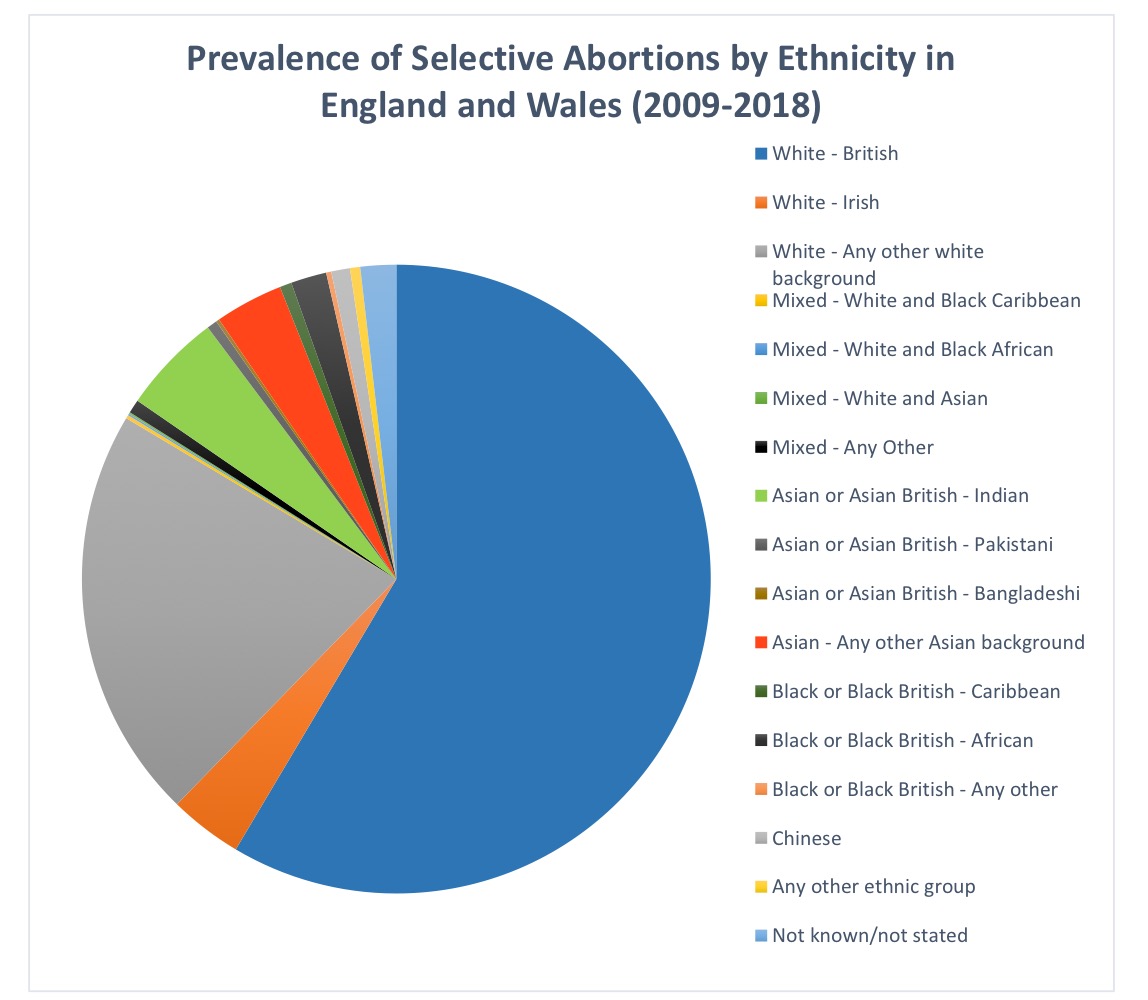


**Supplemental Figure 5.** Prevalence of Selective Abortions by Ethnicity in England and Wales 2009-2018

This graph depicts the prevalence of SA by ethnicity in residents and non-residents of England and Wales between 2009-2018.


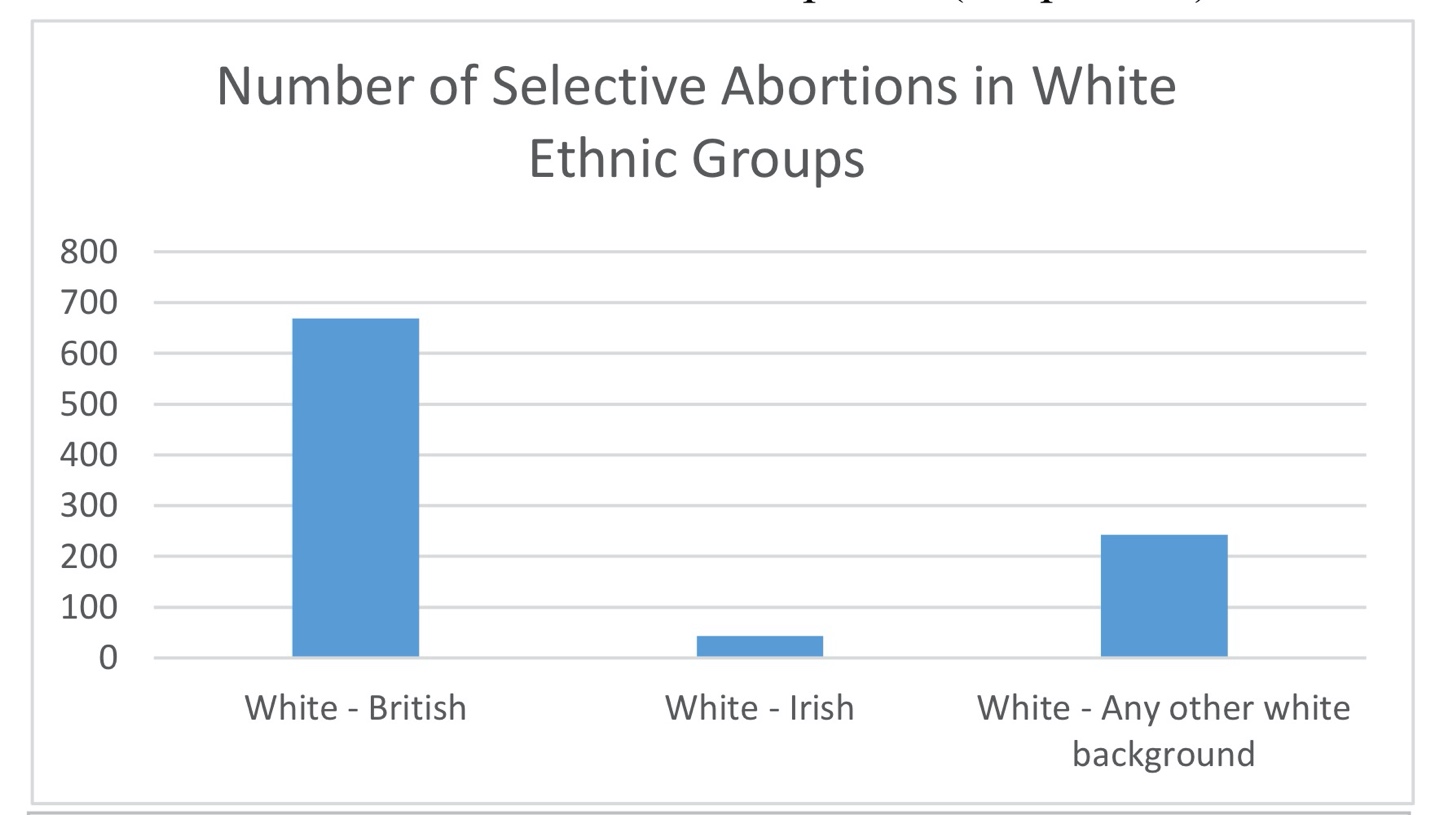


**Supplemental Figure 6.** Number of Selective Abortions in White Ethnic Groups in England and Wales 2009-2018

This graph depicts the breakdown of the prevalence of SAs within White ethnic groups in residents and non-residents of England and Wales (2009-2018). Data from the DHSC.


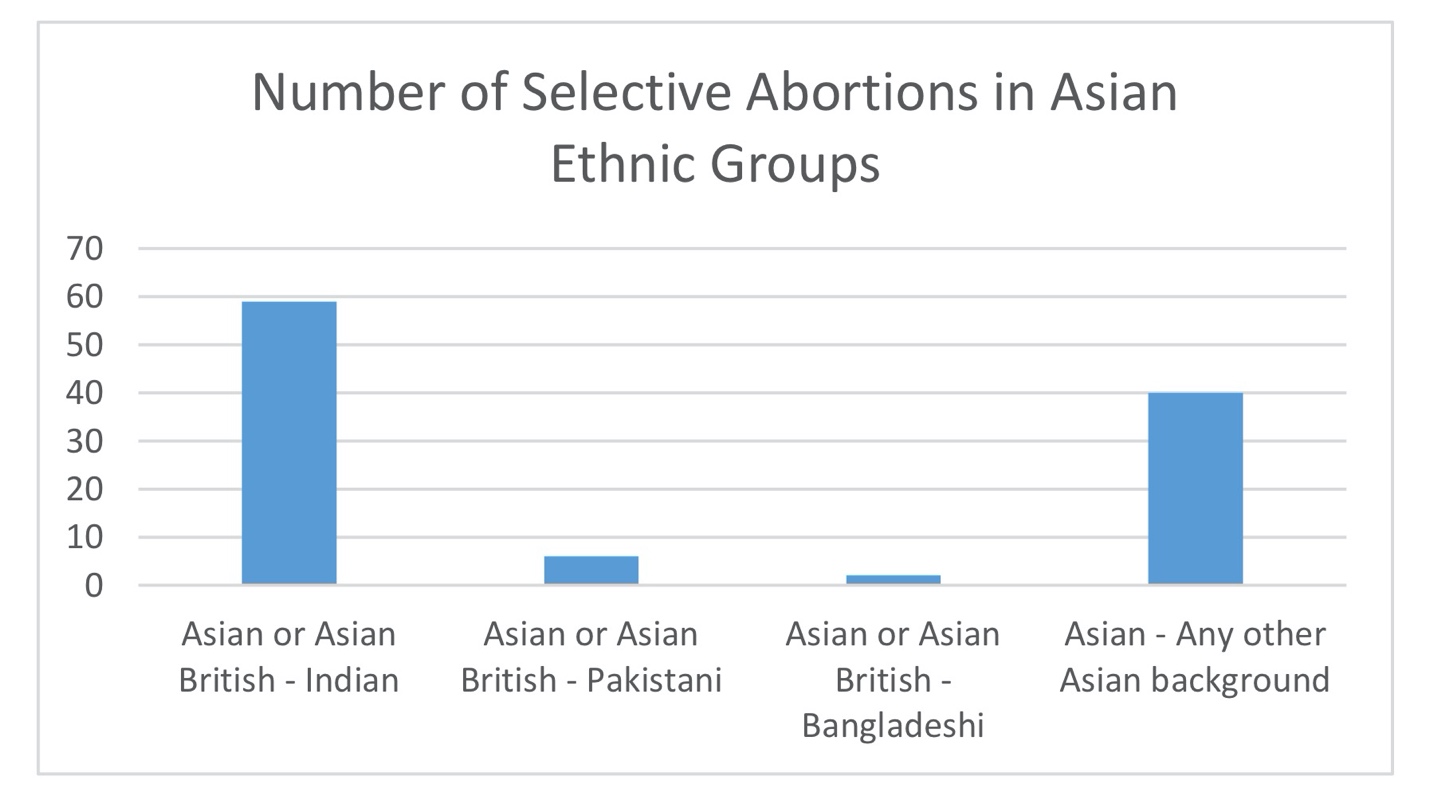


**Supplemental Figure 7**. Prevalence of Selective Abortion in Asian Ethnic Groups in England and Wales 2009-2018

This graph depicts the breakdown of the prevalence of SAs within Asian Ethnic Groups in residents and non-residents of England and Wales (2009-2018). Data from the DHSC.
